# Functional Analysis of Hydrolethalus Syndrome Protein HYLS1 in Ciliogenesis and Spermatogenesis in *Drosophila*

**DOI:** 10.3389/fcell.2020.00301

**Published:** 2020-05-21

**Authors:** Yanan Hou, Zhimao Wu, Yingying Zhang, Huicheng Chen, Jinghua Hu, Yi Guo, Ying Peng, Qing Wei

**Affiliations:** ^1^Laboratory for Reproductive Health, Institute of Biomedicine and Biotechnology, Shenzhen Institutes of Advanced Technology, Chinese Academy of Sciences (CAS), Shenzhen, China; ^2^Chinese Academy of Sciences Key Laboratory of Insect Developmental and Evolutionary Biology, Chinese Academy of Sciences Center for Excellence in Molecular Plant Sciences, Institute of Plant Physiology and Ecology, Chinese Academy of Sciences, Shanghai, China; ^3^University of Chinese Academy of Sciences, Beijing, China; ^4^Department of Biochemistry and Molecular Biology, Mayo Clinic, Rochester, MN, United States; ^5^Institute of Medicine and Pharmaceutical Sciences, Zhengzhou University, Zhengzhou, China

**Keywords:** HYLS1, ciliogenesis, ciliary gate, PCL, centriole elongation, spermatogenesis

## Abstract

Cilia and flagella are conserved subcellular organelles, which arise from centrioles and play critical roles in development and reproduction of eukaryotes. Dysfunction of cilia leads to life-threatening ciliopathies. HYLS1 is an evolutionarily conserved centriole protein, which is critical for ciliogenesis, and its mutation causes ciliopathy–hydrolethalus syndrome. However, the molecular function of HYLS1 remains elusive. Here, we investigated the function of HYLS1 in cilia formation using the *Drosophila* model. We demonstrated that *Drosophila* HYLS1 is a conserved centriole and basal body protein. Deletion of HYLS1 led to sensory cilia dysfunction and spermatogenesis abnormality. Importantly, we found that *Drosophila* HYLS1 is essential for giant centriole/basal body elongation in spermatocytes and is required for spermatocyte centriole to efficiently recruit pericentriolar material and for spermatids to assemble the proximal centriole-like structure (the precursor of the second centriole for zygote division). Hence, by taking advantage of the giant centriole/basal body of *Drosophila* spermatocyte, we uncover previously uncharacterized roles of HYLS1 in centriole elongation and assembly.

## Introduction

Centrioles are small cylindrical cellular organelles typically composed of ninefold symmetric triplet microtubules, which perform two important functions, building centrosome and templating cilia (Bettencourt-Dias and Glover, 2007; Nigg and Stearns, 2011; Conduit et al., 2015). Centrioles recruit pericentriolar material (PCM) to form centrosomes that function as the microtubule-organizing center to organize the mitotic/meiotic spindles. In quiescent cells, centrioles dock to the cell membrane to function as basal bodies to initiate the biogenesis of cilia. Cilia are critical sensory or motile organelles that regulate development and reproduction of eukaryotes (Goetz and Anderson, 2010; Bettencourt-Dias et al., 2011; Malicki and Johnson, 2017; Anvarian et al., 2019). Dysfunction of cilia results in dozens of human genetic diseases, collectively termed ciliopathies (Hildebrandt et al., 2011; Reiter and Leroux, 2017).

In vertebrates, only the mother centriole, but not the daughter centriole, has the ability to form cilia, because only the mother centriole has distal appendage (DA) structures, which is critical for ciliogenesis initiation by mediating centrioles membrane docking. During ciliogenesis, DAs are converted into transition fibers (TFs), one of the conspicuous structures at the cilia base. In invertebrates, such as *Caenorhabditis elegans* (*C. elegans*) and *Drosophila*, centrioles lack DAs, but TFs, at least functional homologous structures, are formed during ciliogenesis. Transition fibers, together with the transition zone (TZ), act as the ciliary gate to control the ciliary protein entry in the context of cilia (Reiter et al., 2012; Takao and Verhey, 2016; Garcia-Gonzalo and Reiter, 2017). The TZ functions as a diffusion barrier to gate the ciliary compartment (Reiter et al., 2012; Garcia-Gonzalo and Reiter, 2017), whereas the TF promotes the import of ciliary proteins. FBF1 is the key TF protein that mediates the entry of ciliary proteins, including intraflagellar transport (IFT) complexes, which are essential for ciliary axonemal elongation (Wei et al., 2013, 2015; Yang et al., 2018). Despite that great progress has been made in understanding the molecular components of DA/TF and their function in ciliogenesis, how such appendages are uniquely assembled from the distal end of mother centriole remains largely as an unsolved mystery (Tanos et al., 2013; Ye et al., 2014; Yang et al., 2018).

Hydrolethalus syndrome (HLS) is a rare recessive lethal inherited disorder that causes serious defects in fetal development and results in birth defects, with its causal gene HYLS1 first identified in 2005 (Mee et al., 2005). Its function was first studied in detail using *C. elegans* as a model organism. Dammermann et al. (2009) found that HYLS1 is a centriole protein that is recruited to the outer centriole wall via direct interaction with the core centriolar protein SAS-4/CPAP. Unexpectedly, HYLS1 is dispensable for centriole assembly, cell division, and embryonic viability in worm, but it is specifically involved in cilia formation in *C. elegans* (Dammermann et al., 2009). Subsequently, we demonstrated that HYLS1 mediates ciliogenesis by regulating the formation of the ciliary gate, plays a major role in recruiting the TF protein FBF1, and plays a minor role in TZ assembly (Wei et al., 2016).

Because of the degeneration of basal bodies after ciliogenesis in *C. elegans* (Sharma et al., 2016), our understanding of how HYLS1 regulates the ciliary gate is limited. Here we sought to address this question using the *Drosophila* model organism. We showed that HYLS1 is a conserved centriole and basal body protein, which is required for ciliogenesis and spermatogenesis in *Drosophila*. Deletion of HYLS1 compromised the localization of ciliary gate proteins in both sensory cilia and spermatocyte cilia. Importantly, by taking advantage of the giant centriole (GC)/basal body in spermatocytes, an excellent model to visualize the dynamic change from centriole to basal body conversion, we found that HYLS1 is critical for the elongation of the GC. In addition, we found that fly HYLS1 is required for efficient PCM components recruitment in spermatocyte and the proximal centriole-like structure (PCL) formation in spermatids. Our findings reveal novel roles of HYLS1 in centriole elongation and assembly during *Drosophila* spermatogenesis, suggesting that the abnormal ciliary gating associated with *hyls1* mutation may arise as a consequential outcome from HYLS1-related distal centriole assembly defects.

## Results

### *Drosophila* HYLS1 Is a Conserved Centriole and Basal Body Protein

Reciprocal protein homology queries using BLAST identified CG42231 as the only annotated gene in *Drosophila* genome with significant homology comparing with mammalian HYLS1 (Supplementary Figure S1). Because CG42231 has never been studied, we named it as HYLS1 hereafter and used genetic manipulations to study its function.

**FIGURE 1:**
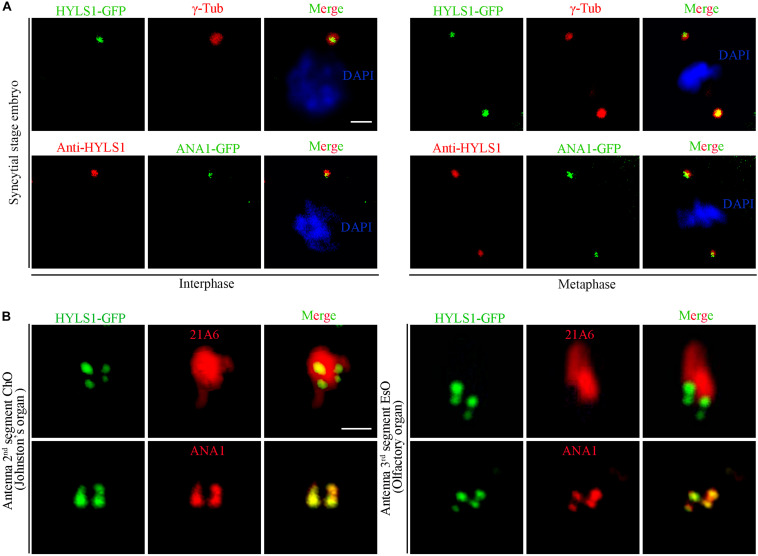
*Drosophila* HYLS1 localizes to centrioles in embryos and basal bodies in sensory cilia. **(A)** In early syncytial stage embryos, GFP-tagged HYLS1 localizes to centrosome labeled by γ-tubulin and centriolar protein ANA1 in both interphase and metaphase. Blue channel in the merged panel represents DNA stained by DAPI. Scale bars: 2 μm. **(B)** Representative images illustrate that HYLS1 is localized to the ciliary base in sensory neurons of both chordotonal organ (ChO) and external sensory organ (EsO). ANA1 is a centriolar protein; 21A6/Eys indicates the base of the sensory cilia. Scale bars: 1 μm.

To determine if the centriole/basal body localization of HYLS1 is conserved in *Drosophila*, we created transgenic flies expressing a green fluorescent protein (GFP) tagged HYLS1 transgene and generated the anti-HYLS1 antibody. The antibody was validated by the following facts: anti-HYLS1 staining showed similar localization pattern as HYLS1-GFP (Figure 1 and Supplementary Figure S2); the staining signal was completely lost in *hyls1* deletion mutants (Figure 3D). As demonstrated by both HYLS1-GFP and anti-HYLS1 staining, HYLS1 colocalized with the centrosome marker γ-tubulin or ANA1 in early syncytial stage embryos of *Drosophila* (Figure 1A and Supplementary Figure S2A), indicating that it is indeed a centriole protein. In *Drosophila*, only Type I monodendritic sensory neurons of the peripheral nervous system (PNS) and sperm cells are ciliated. Type I sensory neurons include external sensory (Es) and chordotonal (Ch) neurons (Jarman, 2002; Boekhoff-Falk, 2005; Kernan, 2007). In both types of ciliated neurons, co-staining of HYLS1-GFP or anti-HYLS1 with the ciliary marker 21A6/EYS (Vieillard et al., 2015) showed that HYLS1 localized to the cilia base (Figure 1B and Supplementary Figure S2B). By colabeling ectopic HYLS1-GFP with the centriole marker ANA1, we observed that HYLS1 completely overlapped with ANA1 in both the distal centriole and the proximal centriole (Figure 1B).

**FIGURE 2:**
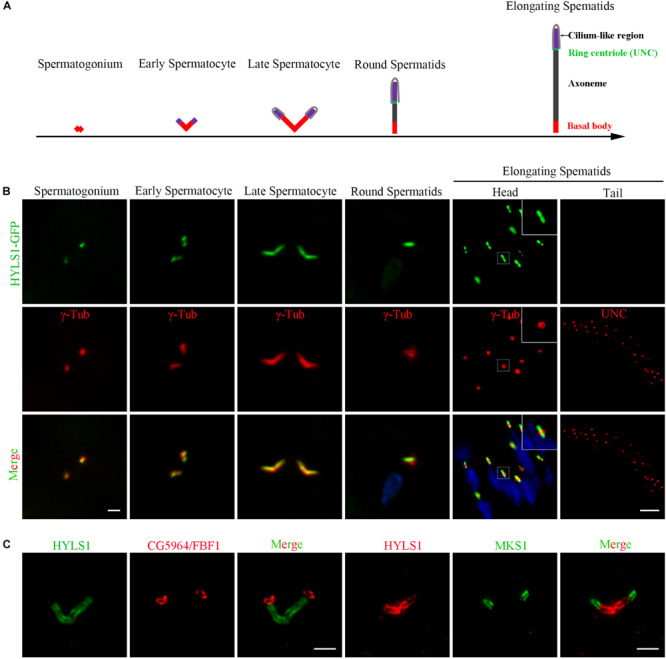
*Drosophila* HYLS1 is a conserved centriole and basal body protein during spermatogenesis. **(A)** Schematic illustration of the dynamic behavior of centrioles during spermatogenesis (see text for details). **(B)** GFP-tagged HYLS1 colocalizes with the centrosome marker γ-tubulin in both spermatogonium and spermatocyte stages. Scale bar: 1 μm. In elongating spermatids, HYLS1 is remained as a basal body protein (magnified region from a head of elongating spermatids, bar: 5 μm), and no HYLS1 migrates to the ring centriole labeled by UNC (bar: 10 μm). Blue channel in the merged panel represents DNA from mature spermatids. **(C)** 3D-SIM images show that HYLS1 distributes along the entire centrioles and below the transition fiber marker FBF1/CG5964 (left) and the transition zone marker MKS1 (right), indicating that HYLS1 is not a TF or TZ component. Scale bars: 1 μm.

In sperm cells, centrioles and basal bodies undergo dynamic changes during spermatogenesis (Fabian and Brill, 2012; Jana et al., 2016; Vieillard et al., 2016; Lattao et al., 2017; Figure 2A). In spermatogonia, centrioles are devoid of appendage structures required for ciliogenesis. In early spermatocytes, centrioles start to accumulate components required for ciliogenesis at their tips. During spermatocyte maturation, paired centrioles dock to the plasma membrane and convert to basal bodies, and then cilium-like structures arise from both the mother and daughter centrioles. Of note, at this stage, paired centrioles/basal bodies elongate dramatically and form GCs/basal bodies with a characteristic V-shaped structure; therefore, it is an attractive model to study the centriole to basal body conversion. In round spermatids, flagellar axonemes start to elongate; cilium-like structures together with ring centriole progressively migrate away from basal bodies. We observed that HYLS1 localized to the centriole in spermatogonia and the basal body in spermatocytes and spermatids, as demonstrated by both HYLS1-GFP and anti-HYLS1 staining (Figure 2B and Supplementary Figure S2C). In spermatocytes, HYLS1 colocalized with γ-tubulin along the whole elongated basal body. In spermatids, HYLS1 presented only at the basal body; no HYLS1 can be detected at the migrated ring centriole marked by UNC (the putative homolog of centriole distal end protein OFD1). Three-dimensional structured illumination microscopy (3D-SIM) showed that HYLS1 localized in a distinctive domain below the TF protein CG5964/FBF1 and the TZ protein MKS1 (Figure 2C). Taken together, we conclude that HYLS1 is a conserved centriole and basal body protein in *Drosophila*.

### *Drosophila* HYLS1 Is Required for Sensory Responses

**FIGURE 3:**
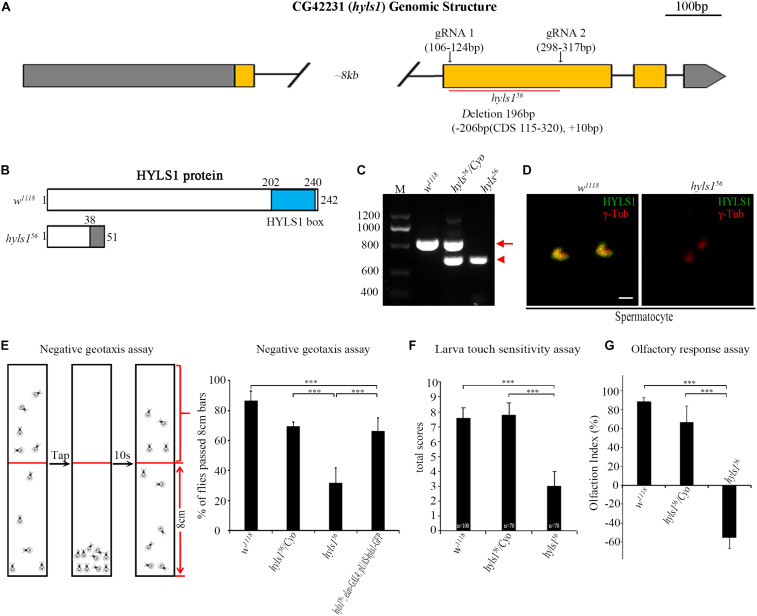
*Drosophila* HYLS1 is required for sensory responses. **(A)** Generation of an *hyls1* null mutant. Diagram shows the genomic region of *Drosophila hyls1* (*CG42231*). CRISPR/Cas9 system was used to create the null mutant *hyls1*^56^. *hyls1*^56^ has a 206-bp nucleotides deletion and an extra 10-bp nucleotides insertion between the targeting sites of gRNA1 and gRNA2 in the second exon. **(B)** Diagram compares the wild-type HYLS1 protein versus the predicted truncated protein. HYLS1 is a 242-residue-long protein, containing a conserved HYLS1 box at its C terminus. *hyls1*^56^ encodes a small, C-terminus truncated protein. **(C)** Genotyping of *hyls1* mutants by PCR, primers are listed in Supplementary Table S1. The size of amplicon from *hyls1*^wt^ is 768 bp (arrow), whereas from *hyls1*^56^ is only of 572 bp (arrowhead). **(D)** Immunostaining by anti-HYLS1 and anti–γ-tubulin confirmed that HYLS1 is lost in the spermatocyte centrosome of *hyls1*^56^ mutants. **(E)** *hyls1*^56^ mutants show reduced climbing ability. The percentage of *hyls1*^56^ flies that passed the 8-cm bars is significantly lower than that in both wild-type and heterozygous *hyls1*^56^/*Cyo* flies. Expression of HYLS1 in PNS neurons with elav-GAL4 rescued the defective phenotype. **(F)** Compared with controls, *hyls1*^56^ homozygous larvae present touch sensitivity defects. Numbers of tested larvae are indicated in the plot. **(G)** Mutation in *hyls1* causes defective olfactory response. Error bars represent mean with SEM. n.s., *p* > 0.05; ***p ≤ 0.001 (Student’s *t-*test). Scale bar: 1 μm.

To understand the function of HYLS1 in *Drosophila*, we employed the CRISPR/Cas9-based genome editing technique to delete most of the second exon of HYLS1 and generated an *hyls1* deletion mutant *hyls1*^56^ (Figures 3A,B). We identified this allele by genomic polymerase chain reaction (PCR) (Figure 3C), and subsequent sequence analysis revealed it bears a 206 bp deletion in the second exon of *hyls1* gene, leading to a predicted reading frame shift. Because only the first 38 amino acids (aa) of the very N-terminus of HYLS1 are predicted to be translated followed shortly by a *de novo* stop codon (Figure 3B), *hyls1*^56^ is likely a null allele. Such assertion was confirmed by a complete loss of endogenous HYLS1 immunofluorescence signal from the basal body in *hyls1* mutants (Figure 3D).

Of note, another gene, *chiffon*, which is involved in DNA replication and histone acetylation (Landis and Tower, 1999; Torres-Zelada et al., 2019), is in the intron between the exon 1 and exon 2 of *hyls1*. *chiffon* has four annotated isoforms: RB and RD have no shared exons with *hyls1* and encode the same 1,695-aa protein; RE encodes the shortest protein of 576 aa residing within RB/RD; RA encodes a 1,711-aa protein. The extra 30-aa C-terminus of RA is encoded by a quite short exon shared with part of exon 2 of *hyls1*, which is deleted in our *hyls1*^56^ mutants (Supplementary Figure S3). As the 1,695-aa protein is fully functional (Torres-Zelada et al., 2019), our mutant should not affect the function of chiffon. Such conclusion was further supported by the fact that our *hyls1*^56^ mutant flies are viable and females are fertile, whereas deletion of CHIFFON results in female infertility and adult lethality (Landis and Tower, 1999; Torres-Zelada et al., 2019).

*hyls1*^56^ mutants show moderately uncoordinated phenotype (Supplementary Videos S1, S2), a phenotype usually associated with cilia mutants. Interestingly, the uncoordinated phenotype was fully rescued by expression of a WT *hyls1* transgene in neurons driven by *elav*-GAL4, confirming that HYLS1 is the gene responsible for behavior defects in *hyls1*^56^ mutants (Supplementary Video S3).

First, we examined the cilium ultrastructure of JO in *hyls1* mutants using transmission electron microscopy. JO is composed of a various number of scolopidia. A scolopidium usually consists of two neurons with cilia residing on the dendrite tip. In WT, two axonemes are present in the cross section of a scolopidium. However, in *hyls1* mutants, missing axoneme was observed (Supplementary Figure S4), indicating that a subset of cilia is truncated in *hyls1* mutants.

As cilia are essential for sensory response in neurons, we then examined the sensory abilities of *hyls1* mutants with a series of behavioral assays. Negative geotaxis has been well characterized in flies, governed by ciliated sensory organs, which are responsible for gravity perception and locomotor coordination (Kernan, 2007). Wild-type flies climb upward against gravity, after tapping down to the bottom of a tube; most of them move back toward the top in a few seconds. On the contrary, most *hyls1* mutants stayed at the bottom of the testing tube after tapping, indicating that negative geotaxis behavior is significantly affected in *hyls1* mutants (Figure 3E). Accordingly, climbing defect was effectively rescued by expression of a WT *hyls1* transgene in neurons driven by *elav*-GAL4, confirming that HYLS1 is indeed responsible for negative geotaxis defect in mutant flies. To test the mechanosensory capabilities of *hyls1* mutants, we performed touch sensitivity assay and found the response to touch in larva of *hyls1* mutants was also severely impaired (Figure 3F). Moreover, we conducted the Y-maze assay to assess the olfactory sensitivity of *hyls1* mutants. As expected, *hyls1* is required for normal chemical sensation because *hyls1* mutants have serious defects in attraction by grape juice. Instead, it seems that *hyls1* mutants reject grape juice (Figure 3G). This result indicates that not only the normal olfactory response is disturbed, but also the response pathway also undergoes some changes, which may be due to simultaneous defects in the transport of certain membrane proteins and/or signal molecules that promote or inhibit olfactory function. Altogether, these behavioral assays demonstrate that HYLS1 is required for neuron sensory response in *Drosophila*.

### *Drosophila* HYLS1 Is Required for Protein Trafficking in Sensory Cilia

**FIGURE 4:**
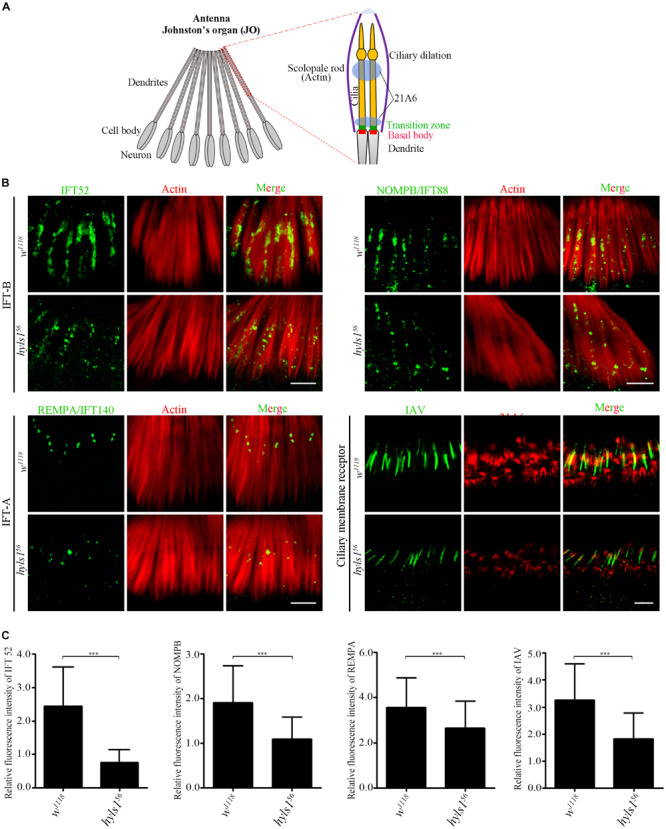
HYLS1 is required for the entry of ciliary proteins in *Drosophila*. **(A)** Schematic view of the Johnston’s organ (JO) at the second antennae segment. JO is a specialized chordotonal organ (ChO), which is composed of a various of scolopidia. A scolopidia consists of two Ch neurons and several accessory cells. Ch neurons bear cilia from their dendrite tip; details of the ciliary region of a single scolopidia are illustrated on the right panel. Actin marks scolopale rods, which enclosed the cilia, and 21A6 labels the scolopale lumen around the proximal end of the cilia and the region below the ciliary dilation. **(B)** HYLS1 is required for the ciliary entry of IFT components in *Drosophila*. The ciliary signal intensities of IFT-B components IFT52 and NOMPB/IFT88, IFT-A protein REMPA/IFT140, and ciliary membrane protein IAV are significantly reduced in *hyls1* mutants. Bars: 5 μm. **(C)** Quantifications of relative fluorescence intensities of IFT52 (control *n* = 89; *hyls1*^56^ *n* = 85), NOMPB (control *n* = 91; *hyls1*^56^ *n* = 53), REMPA (control *n* = 148; *hyls1*^56^ *n* = 125), and IAV (control *n* = 132; *hyls1*^56^ *n* = 132) in WT and *hyls1* mutants. Error bars represent ± s.d., n.s., *p* > 0.05; ***p ≤ 0.001 (Student’s *t-*test). Scale bars: 2 μm. “n” is the number of cilia examined.

It has been reported that HYLS1 is required for the function and formation of ciliary gate (Wei et al., 2016). Therefore, we first examined the localization of ciliary soluble proteins (IFT components) and membrane protein (IAV) in cilia of *hyls1*^56^ mutants. Axonemes of auditory cilia in the second antennal segment are longer, which are more suitable for cilia study. As expected, we observed that compared with WT, the ciliary level of IFT-B components IFT52 and NOMPB (the ortholog of human IFT88) and the IFT-A components REMPA (the ortholog of human IFT140) was significantly reduced in auditory cilia of *hyls1*^56^ mutants (Figure 4B). These observations were further confirmed by our quantitative analysis of the signal intensity of these IFT proteins or the ratio of IFT protein intensity to the actin intensity in the associated scolopidia (Figure 4C and Supplementary Figure S5). Accordingly, the amount of ciliary membrane receptor IAV was also dramatically reduced in sensory cilia of *hyls1*^56^ mutants (Figures 4B,C). These results indicate that deletion of HYLS1 compromised the entry of ciliary proteins.

**FIGURE 5:**
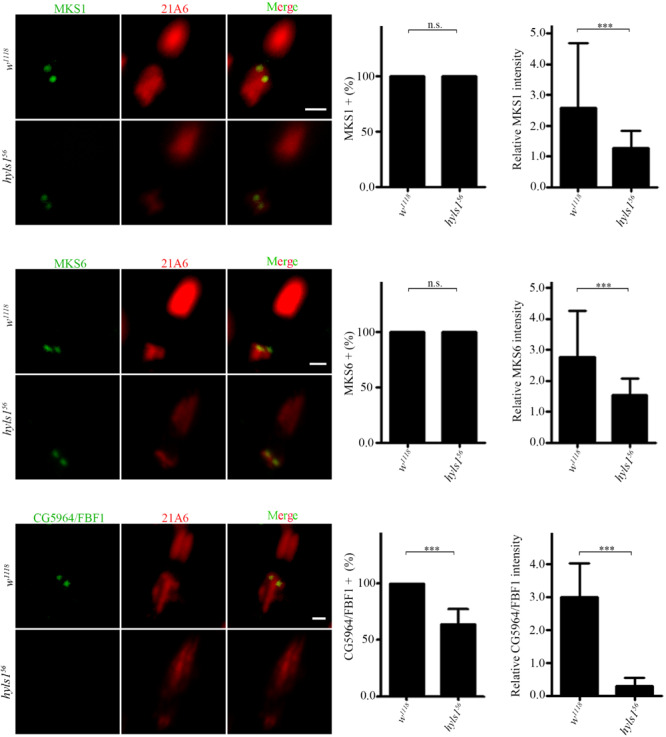
HYLS1 is required for the proper localization of ciliary gate proteins in sensory cilia in *Drosophila*. Representative images of ciliary gate proteins MKS1 (control n = 115; *hyls1*^56^ *n* = 70), MKS6 (control n = 69; *hyls1*^56^ *n* = 105), and CG5964 (control *n* = 98; *hyls1*^56^ *n* = 59) in WT and *hyls1* mutants. Corresponding quantifications of relative fluorescence intensities were shown in the right panel. Error bars represent ± s.d., n.s., *p* > 0.05; ***p ≤ 0.001 (Student’s *t*-test). Scale bars: 2 μm. “n” is the number of cilia examined.

Then, we determined ciliary gate formation in *hyls1*^56^ mutants by examining the localization of ciliary gate proteins. The TZ, together with TFs, forms the ciliary gate (Reiter et al., 2012; Takao and Verhey, 2016; Garcia-Gonzalo and Reiter, 2017). As shown in Figure 5, the signals of TZ protein MKS1 and MKS6 were dramatically reduced in *hyls1*^56^ mutants compared to WT, although they were still able to target to the basal body. Transition fiber structures have been observed at the base of sensory cilia in *Drosophila* (Jana et al., 2018). FBF1 is the key TF protein mediating the ciliary protein entry in nematodes and mammals. Its *Drosophila* homolog is encoded by CG5964, and we have characterized CG5964 as a functional TF protein in *Drosophila* (Y.H., unpublished results). Interestingly, consistent with what we have reported in *C. elegans*, CG5964 was completely lost or only minimally retained in sensory cilia in *hyls1* mutant flies (Figure 5). Taken together, our results suggest that HYLS1 is involved in the function and formation of the ciliary gate in *Drosophila*.

### HYLS1 Is Essential for Giant Centriole Elongation in Spermatocytes

**FIGURE 6:**
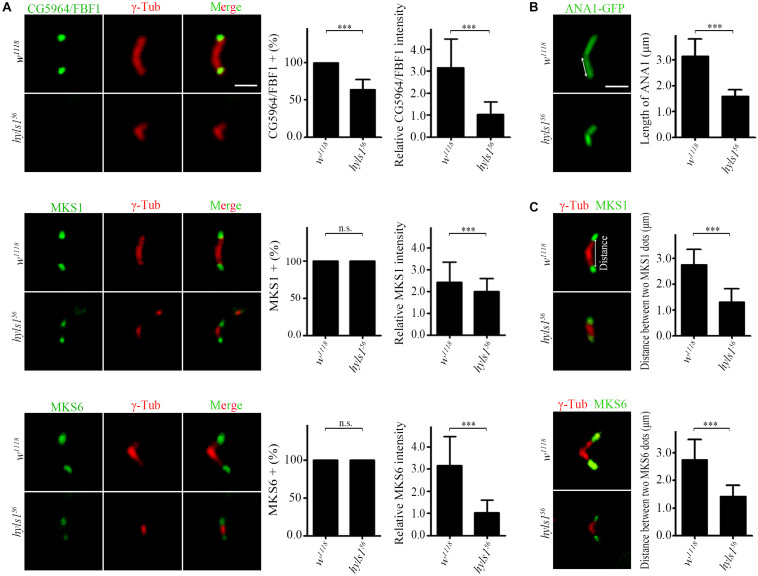
HYLS1 is essential for giant centriole elongation in spermatocytes. **(A)** In *hyls1*, CG5964 partially failed to be recruited to centriole tips in spermatocytes (control *n* = 87; *hyls1*^56^ *n* = 57; upper), and reduced signal intensities of MKS1 (*n* = 143; *hyls1*^56^ *n* = 76; middle) and MKS6 (control *n* = 103; *hyls1*^56^ *n* = 98; lower) were observed. **(B)** Representative micrographs of meiosis II centrioles stained by ANA1-GFP (right) in testes from control and *hyls1* mutants. Quantification of centriole length was shown in the right panel. **(C)** Deletion of HYLS1 leads to shorter distance between two transition zone dots associated with V-shaped centrioles (upper, MKS1; lower, MKS6). The relative distance between two signal dots (double-headed arrow) was quantified in the right panel. Scale bars: 2 μm. For all charts, error bars represent ± s.d.; statistical analysis was done with paired Student’s *t-*test, n.s., *p* > 0.05; ***p ≤ 0.001.

Next, we examined the role of HYLS1 in spermatocyte cilia, which have no IFT and only contain conserved TZ proteins (Han et al., 2003; Sarpal et al., 2003; Williams et al., 2011). Although TF structures have not been observed in spermatocyte cilia (Riparbelli et al., 2012; Jana et al., 2018), CG5964 is indeed localized to a distinct region corresponding to the TF, at the tip of centriole and below the TZ. Consistent with what we observed in sensory cilia, the signal intensities of CG5964 and TZ proteins MKS1 and MKS6 were significantly reduced in spermatocyte cilia of *hyls1* mutants compared to WT (Figure 6A). And again, CG5964 is more sensitive to HYLS1 deletion. In approximately 30% of cilia, CG5964 was completely lost.

Surprisingly, we observed that, compared with WT that has a large V-shaped centriole pair, the centrioles in *hyls1* mutant spermatocytes were significantly shorter, as indicated by the centriole marker ANA1/CEP295 (Figure 6B). This phenotype is specific to the centriole elongation, because TZ proteins can still be recruited to the tip of growing centrioles throughout spermatocytes development. To further confirm the role of HYLS1 in regulating centriole elongation, we measured the distance between two TZ protein dots associated with a given pair of centrioles. As expected, the results showed that the mean distance between two protein dots in *hyls1* mutants was significantly shorter than that in WT (Figure 6C).

As a centriole protein, how could HYLS1 regulate the localization of TF/TZ proteins? One possible explanation is that HYLS1 regulates the elongation/assembly of the distal centriole where TF/TZ are localized. However, no supportive evidence has been reported. Our observations provide the strong evidence for this hypothesis. We suggest that defects in centriole elongation contribute to defects in TZ and TF in *hyls1* mutants. Without proper HYLS1-dependent centriole growth and structural remodeling, TF and TZ can be formed but with significant abnormalities (such as decreased localization of TF and TZ proteins).

### *Drosophila* HYLS1 Is Required for PCM Recruitment in Spermatocytes and PCL Formation in Spermatids

**FIGURE 7:**
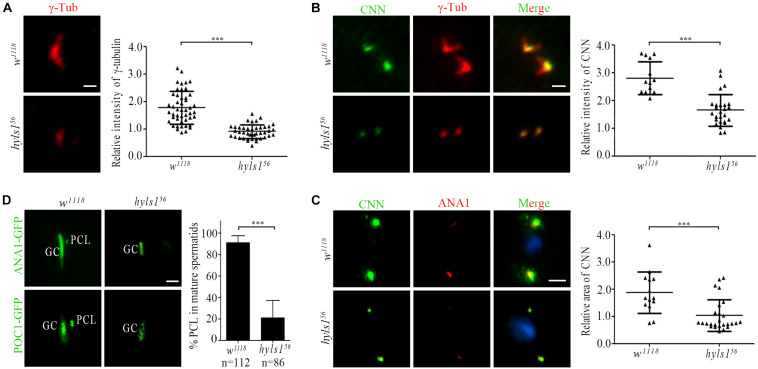
*Drosophila* HYLS1 is required for PCM recruitment in spermatocytes and PCL formation in spermatids. **(A)** γ-Tubulin signal was significantly reduced in spermatocyte centriole in *hyls1*^56^. Quantification data of the relative signal intensity are shown in the right panel. **(B)** HYLS1 is required for recruitment of PCM protein CNN in interphase of spermatocyte. The right panel shows the quantification of relative centrosome fluorescence intensities of CNN in controls and *hyls1* mutants. **(C)** Images of CNN signal and corresponding quantification data (right panel) in metaphase of meiosis in controls and *hyls1* mutants. Blue channel in all panels represents DNA from meiotic chromosomes. **(D)** Deletion of HYLS1 results in abnormal PCL formation indicated by ANA1-GFP and POC1-GFP. GC, giant centriole; PCL, proximal centriole-like structure. Scale bars: 1 μm (A, B, D), 5 μm **(C)**. Scattered plots with mean and SD are shown. n.s., *p* > 0.05; ***p ≤ 0.001 (Student’s *t-*test).

In addition to the short centrioles, we observed that the signal of γ-tubulin was dramatically reduced in centrosomes in spermatocytes of *hyls1* mutants compared to WT (Figure 7A). Because γ-tubulin is a PCM component, we examined the level of another PCM component CNN. Compared with WT, the amount of CNN was significantly decreased in interphase in *hyls1* mutant cells as well (Figure 7B). Pericentriolar material enlarges in meiosis, and its component CNN is robustly recruited to the centrosome. Quantification of the CNN signal revealed that the recruitment of CNN around HYLS1-negative meiotic centrosomes was significantly less robust comparing with WT (Figure 7C). All these results collectively indicated that HYLS1 is involved in PCM recruitment in spermatocytes.

*Drosophila* spermatids have two centrioles: one is a typical centriole (GC), and the other one is an atypical centriole (PCL) adjacent to the distal part of GC (Blachon et al., 2009). Both centrioles are required for zygote mitosis (Gottardo et al., 2015; Khire et al., 2016). Strikingly, we observed that, in addition to the short typical centriole, PCL was often lost in *hyls1* mutant spermatids. We confirmed this result by using two PCL marker proteins ANA1 and POC1 (Figure 7D). It is not likely that HYLS1 is a PCL protein, as we did not observe that HYLS1 localizes to PCL (Figure 2B and Supplementary Figure S2B). Given the fact that PCL is adjacent to the distal part of GC, it is tempting to speculate that PCL formation depends on centriole distal part; loss of PCL in *hyls1* mutants may be caused by the defects in centriole elongation.

Taken together, our results indicate that HYLS1 is required for centriole elongation and assembly in *Drosophila* sperm cells.

### HYLS1 Is Required for Spermatogenesis in *Drosophila*

**FIGURE 8:**
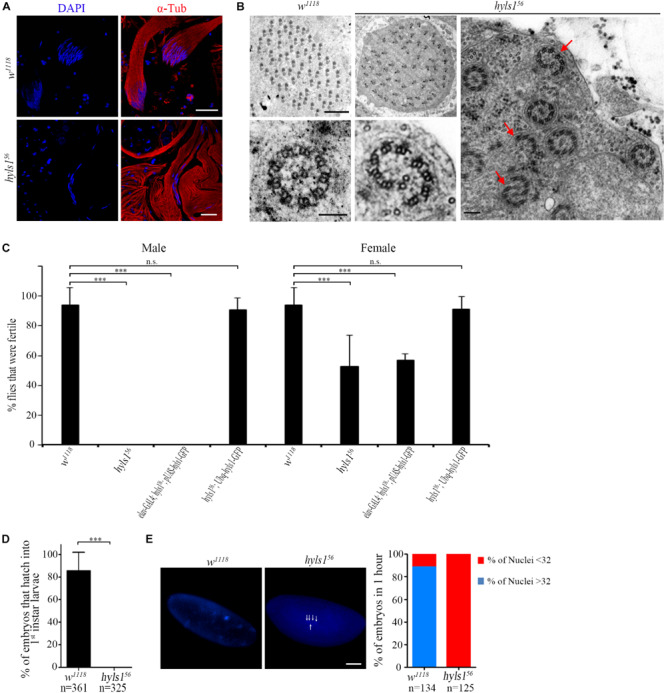
HYLS1 is required for spermatogenesis in *Drosophila*. **(A)** Cysts of elongating spermatids in wild-type (upper) and *hyls1* mutant (lower) testis stained by nuclei marker DAPI (blue) and sperm flagellar marker α-tubulin (red). Nuclei were clustered in WT cysts but were abnormally dispersed in *hyls1*^56^ mutants. **(B)** Ultrastructure of sperm flagellar axoneme of WT and *hyls1*^56^ mutants showed that the spermatids in the *hyls1*^56^ cyst were irregularly arranged, and some axonemes were incomplete or broken (red arrows). **(C)** *hyls1*^56^ males are completely infertile. Introduction of HYLS1 in neurons driven by elav-Gal4 could not rescue the male fertility. Fertility is fully recovered with global HYSL1 rescue (*Ubq-hyls1-GFP*). Mutations in *hyls1* also affected female fertility. **(D,E)** HYLS1 is essential for embryogenesis. Embryos fathered by *hyls1* mutant failed to develop into larva **(D)** and were arrested in the early development stage **(E)**. Scale bars: 20 μm in **(A)**, 2 μm in (**B** left), 100 nm in (**B** right), 100 μm in **(E)**. Error bars represent ± s.d., n.s., *p* > 0.05; ***p ≤ 0.001 (paired Student’s *t-*test).

To determine if HYLS1 is required for spermatogenesis, we examined sperm production. In *hyls1* mutants, mature sperms were accumulated in the seminal vesicles and were motile; no visible abnormalities were observed. Colabeling of DNA and α-tubulin in spermatocyte cyst showed that spermatids elongate in *hyls1* mutants. Surprisingly, in wild type, 64 flagella tightly associate with each other to form a well-organized bundle, whereas in *hyls1* mutants, nuclei were dispersed, and the bundled organization of spermatids was severely disordered (Figure 8A). When examining the sperm flagella axonemal ultrastructure by electron microscopy, we frequently observed incomplete and broken axonemal in *hyls1* mutants (32.5%; *n* = 471; Figure 8B; red arrow), and 8 of 19 (42.15%) cysts had 63 or fewer spermatids, whereas all control cysts possessed 64 spermatids (Figure 8B). Considering that sperm flagella formation is independent of IFT in *Drosophila*, we speculated that the role of HYLS1 in basal body might directly contribute to defects in spermatogenesis.

Although only moderate sperm defects in *hyls1* mutants, homozygous *hyls1* males are completely infertile (Figure 8C). No zygotes derived from *hyls1* mutant males could successfully develop into larvae even when homozygous mutant males were mated to wild-type (*w*^1118^) females (Figure 8D). Male fertility can be fully rescued by a ubiquitously expressed WT *hyls1* transgene (*Ubq-hyls1-GFP*), but not by restricted rescue of HYLS1 in neurons (*elav-GAL4; pUAS-hyls1-GFP*), indicating that infertility is not likely due to the impaired sensory or courtship capacity but owing to defects in spermatogenesis and/or embryogenesis after fertilization (Figure 8C). Further studies revealed that the development of embryos fathered by *hyls1*^56^ was dramatically delayed and arrested at the early cleavage stage of *Drosophila* embryos. Nearly 85% embryos have more than 32 nuclei in 1-hour-old control embryos, whereas no embryos with more than 32 nuclei were observed in 1-hour-old *hyls1* embryos (Figure 8E). Because both GC and PCL of spermatids are required for zygote mitosis, it is tempting to speculate that defects in GC and PCL may also contribute to embryonic development arrest in paternal *hyls1* mutants. Of note, female *hyls1* flies were partial fertile, and the phenotype was fully rescued by expressing a *Ubq-hyls1-GFP* transgene but not a *pUAS-hyls1-GFP* only in nervous system (Figure 8C), suggesting that maternal HYLS1 may also play a role in embryogenesis. More works will be needed to understand the role of HYLS1 in embryogenesis in the future.

## Discussion

Here we investigated the function of HYLS1 in ciliogenesis and spermatogenesis in *Drosophila*. We demonstrated that the centriole/basal body localization of HYLS1 and its role in ciliary function are highly conserved in *Drosophila*. More importantly, by taking advantage of the GC in *Drosophila* spermatocytes, we demonstrated that *Drosophila* HYLS1 is required for centriole elongation during spermatogenesis.

Although conventional TF structures observed in mammalian cells may not be conserved in *C. elegans* and *Drosophila*, the presence of conserved components suggests that, at least, alternative functional homologous structures should exist (Ye et al., 2014; Yang et al., 2018). In both *C. elegans* and *Drosophila*, HYLS1 is required for the localization of key TF protein FBF1. As a centriole protein, how could HYLS1 regulate the localization of a TF protein? One highly possible explanation is that HYLS1 regulates the elongation of the distal centriole required for TFs anchoring. However, short or degenerated basal body limited the analysis of centriole elongation during centriole to basal body conversion in sensory cilia in both *C. elegans* and *Drosophila*. Remarkably, by taking advantages of the GC in *Drosophila* spermatocytes, we do observe that HYLS1 promotes centriole elongation. Our observation leads us to propose that HYLS1 is required for distal centriole elongation and recruiting distal centriole proteins to build a specialized construction stage to support the *de novo* assembly of TFs. Without HYLS1, such stage is compromised, which has direct structural and functional consequences toward the associated appendages made at the distal tip of growing centriole. Although our model is largely based on observations made on the growth of specialized centrioles (*Drosophila* spermatocytes GCs), we speculate similar HYLS1-dependent distal centriole extension process is a general prerequisite for cilia formation based on the mother centriole. Verification of such generalization calls for further refinement of super-resolution molecular imaging techniques to resolve the full dynamics of cilia formation.

What is the possible role of HYLS1 in centriole elongation or assembly? It has been reported that HYLS1 interacts with the core centriolar protein SAS-4 (Dammermann et al., 2009). Because SAS-4 binds tubulin and is essential for centriole elongation and assembly, it is highly possible that HYSL1 functions through SAS-4 to regulate centriole elongation. In addition, SAS-4 cooperates with CEP120 and SPICE1 to regulate centriole elongation (Comartin et al., 2013; Lin et al., 2013); it is also possible that HYLS1 serves as a critical mediator bridging interactions between SAS-4 and other regulators of centriole elongation. Of note, SAS-4 is also required for PCM assembly (Gopalakrishnan et al., 2011, 2012; Zheng et al., 2014). It is also possible that HYLS1 functions through SAS-4 to regulate PCM recruitment. It will be interesting to test all these hypotheses in the future.

In *C. elegans*, HYLS1 is not required for centriole assembly and centrosome function (Dammermann et al., 2009). In *Drosophila*, the development of *hyls1* mutant is grossly normal, suggesting that HYLS1 may be also dispensable for centriole and centrosome function in tissues other than sperm cells. In both *C. elegans* and *Drosophila*, centrioles are short and lack of appendage structures and probably lack of distal structures found in vertebrate centrioles. Because the distal part of the mammalian centriole is usually not important for centriole duplication and assembly, but is critical for ciliogenesis, it is tempting to speculate that HYLS1 mainly plays a role in the elongation or assembly of the centriole distal part. In conclusion, our finding advances understanding on HYLS1 function and suggests that not only cilia but also the HYLS1-dependent centrosome remodeling may directly contribute to the pathogenesis of human HLS.

## Materials and Methods

### Fly Stocks

All flies were cultured on standard media at 18°C or 25°C. *w*^1118^ was used as wild-type fly. The following flies were obtained from Bloomington Drosophila Stock Center: *elav-GAL4* (BS458; BDSC). Mks1-GFP and Mks6-GFP strains were described previously (Vieillard et al., 2016) and were gifts from Bénédicte Durand. IAV-GFP, RempA-YFP, and NompB-GFP were gifts from Seok Jun Moon (Park et al., 2013). All the other following transgenes were generated in the laboratory: *Ubq-hyls1-GFP*, *pUAS-hyls1-GFP*, *Ubq-ana1-GFP*, *Ubq-cnn-GFP*, *Ubq-poc1-GFP*, *CG5964-GFP*, *unc-GFP*, and *ift52-GFP*.

### Transgenic *Drosophila* Lines

To generate ubiquitin-driven transgenic lines, promoter of ubiquitin was inserted into the *Hin*dIII site of the pJFRC2 vector^1^ first, and then CDS sequences of HYLS1, POC1B, and CNN-PA were cloned into the *Not*I–*Bam*HI site of the pJFRC2 plasmid in frame with the downstream GFP sequence. To make *CG5964-GFP*, *unc-GFP* and *ift52-GFP* transgenic lines, their coding, and upstream regulatory sequences were amplified from genomic DNA and then cloned into the *Hin*dIII–*Bam*HI site of the pJFRC2 plasmid in frame with the downstream GFP sequence.

To get *pUAS-hyls1-GFP* flies, full-length HYLS1 CDS was cloned into *Not*I–*Bam*HI digested pJFRC14 vector.

All vectors were injected into *Drosophila* embryos by the Core Facility of *Drosophila* Resource and Technology, SIBCB, CAS. The resulting transgenic flies were selected according to standard procedure.

All GFP signals in colabeling immunofluorescence assay were stained with anti-GFP.

All DNA fragments used were amplified from genomic DNA by us. All CDS fragments were amplified from cDNA reverse transcribed from our own extracted total RNA. All primers used are listed in Supplementary Table S1.

### Generation of *hyls1* Mutant

We used the CRISPR/Cas9 technique to generate the *hyls1* knockout mutants. Two gRNAs were selected using Target Finder website^2^, gRNA 1: 5′-CGCAAGCTCATCAAGCATGAGG-3′ and gRNA 2: 5′-CTTAAGGAGCGCTCCGATGGTGG-3′. gRNA sequences were separately cloned into a modified pEASY-Blunt vector with a U6.3 promoter. Two gRNA plasmids were collectively injected into vasa:Cas9 embryos, and deletion lines were screened with genomic PCR and were outcrossed five times to wild-type flies (*w*^1118^).

### Immunofluorescence and Microscopy

#### Embryo Staining

Embryos were dechorionated in 50% bleach for 5 min to remove chorion, and vitelline membranes were gently stripped under the stereomicroscope and then fixed in a mixture of heptane and 4% formaldehyde (1:1) for 30 min with shaking. The fixed embryos were washed in phosphate-buffered saline (PBS) and blocked with 3% bovine serum albumin (BSA) in PBS-T (PBS 1 × + 0.1% Triton X-100) for 1 h and then incubated with primary antibodies for 24 h at 4°C. The samples were then washed three times with PBS-T for 15 min each and then incubated with secondary antibodies for 3 h at room temperature. After washing three times with PBS-T, embryos were then mounted on slides.

#### Whole-Mount Antennae Staining

Antennae from pupal or 1- or 2-days-old flies were dissected in PBS-T (PBS 1 × + 0.3% Triton) and then fixed in 4% paraformaldehyde in PBS-T for 1 h at room temperature. The fixed antennae were then incubated in blocking solution (PBS 1 × + 0.1% BSA + 0.1% Triton X-100) for 1 h and stained with primary antibody for 48 h and then secondary antibodies for 24 h.

#### Testis Squashes Staining

Testes from young males (1 or 2 days old) or pupae were dissected in PBS and then were squished between cover glass and microscopy slide. After squashing, the samples were snap-frozen in liquid nitrogen, and then the coverslips were removed off, and the slides were immersed immediately into methanol for 10 min at –20°C and sequentially in acetone for 10 min at –20°C. Slides were washed in PBS-T and blocked with 3% BSA in PBS-T for 1 h and then were incubated with primary antibodies overnight at 4°C and secondary antibodies for 3 h at room temperature. Lastly, testes were mounted and examined using fluorescence microscopy.

### Antibodies

The following primary antibodies were used: mouse anti–γ-tubulin (1:500; Sigma-Aldrich, St. Louis, MO, United States), mouse anti–α-tubulin (1:500; Santa Cruz Biotechnology, Dallsa, TX, United States), mouse anti-GFP (1:500; Roche, Basel, Switzerland), rabbit anti-GFP (1:500; Abcam, Cambridge, United Kingdom), mouse 22C10 (1:200; DSHB), mouse 21A6 (1:200; DSHB), iFluor^TM^ 555 Phalloidin (1:200; Yeasen, Shanghai, China). The following secondary antibodies were used: goat anti–mouse Alexa Fluor 488 or Alexa Fluor 594 or goat anti–rabbit Alexa Fluor 488 or Alexa Fluor 594.

### Generation of *Drosophila* HYLS1 Antibody

All the work of generation of HYLS1 antibody is done by YOUKE Biotech, Shanghai, China. Briefly, His-tagged full-length HYLS1 protein was expressed in *Escherichia coli* and purified as immunogen. Antisera were raised in two rabbits and purified by protein G beads; 1:200 dilution was used for immunofluorescence assay.

### Microscopy

Samples were imaged using either the fluorescence microscopy (Nikon Eclipse Ti, Nikon, Japan) or the confocal microscopy (Olympus FV1000a, Olympus, Japan) with a 100 × /NA 1.45 oil immersion objective. For super resolution imaging, samples mounted in ProLong^TM^ Diamond Antifade Mountant (Invitrogen, Carlsbad, CA, United States) were imaged using a 3D-SIM (Delta Vision OMX SR, GE Healthcare, Chicago, IL, United States).

### Quantification of Fluorescence Intensity

To minimize the staining variation between mutant and control, both samples were prepared and stained at the same time and mounted on a same slide, and fluorescence images were obtained under the same microscopic conditions and settings. The fluorescence intensity was measured using Nikon NIS-Elements software, and background fluorescence was subtracted.

### Transmission Electron Microscopy

Dissected fly antennae and testes were immediately held in 2.5% glutaraldehyde for at least 24 h before postfixation in OsO_4_. Then samples were dehydrated in alcohol and then embedded in epoxy resin. Sections of (∼70 nm) of samples were cut in Ultracut S ultramicrotome, collected on Formvar-coated copper grids, and stained with uranyl acetate and lead citrate. Samples were imaged with H-7650 electron microscope (Hitachi, Japan) at 80 KV.

### Fertility Ratio Assay

For male fertility ratio assay, virgin *w*^1118^ females and mutant males are crossed. For female fertility ratio assay, mutant females and *w*^1118^ males are crossed. Before testing, virgin females and newly enclosed males were separately collected and held in room temperature for 3–4 days. In each test, a single male was mated individually with a single virgin female for 4 days at 25°C. Crosses with dead flies were eliminated from the test. Fertility ratio was quantified.

### 24 h Embryo Development Assay and Larval Hatching

Virgins and males of 3–4 days were placed in a mating chamber at 25°C to lay eggs. Parents were removed until more than 80 embryos were laid, and embryos were cultured at room temperature for 3 days. The percent of hatched eggs was counted. Experiments were repeated at least three times.

### Negative Geotaxis Assay (Climbing Assay)

Climbing assay was performed as described previously (Chen et al., 2015). Briefly, 10 flies were transferred as a group to a fresh food vial the day before the test. Just before the experiment, flies were transferred into a 20-cm-long clear testing vial. During the test, flies were gently tapped down to the vial bottom; the flies climbing above the 8-cm mark were counted after 10 s. After the test, flies were given 1 min to recover, repeated 10 times. At least five groups of flies were assessed.

### Larva Touch Assay

Larva touch assay was performed as previously described (Chen et al., 2015). Briefly, 10 larvae as a group were gently touched one by one on their head segments with a human hair. According to the response of the larva to the touch, a score was assigned: 0 for the larva showed no response; 1 for the larva showed hesitation with ceased movement; 2 for the larva that showed anterior contract; 3 for one full wave of body contraction; and 4 for the larva that showed two or more full waves of body contractions. Each group was tested four times, and the four scores were added up to a total score; at least five groups of larvae were assayed.

### Olfactory Response Assay

Olfactory response was tested by using the Y-maze assay as described (Simonnet et al., 2014). Briefly, before testing, 10 flies as a group were starved for 16–18 h at 25°C in a glass tube. Just before the test, a small filter paper with odorant or corresponding solvent was placed into the two trap vials, respectively, and flies were loaded into the loading vial. Olfactory index was calculated after 24 h. At least five groups of flies were tested each experiment.

## Data Availability Statement

The datasets generated for this study are available on request to the corresponding author.

## Author Contributions

QW and YH designed the study and analyzed the data. YH performed the experiments. ZW, YZ, and HC assisted the experiments and performed the SIM imaging. YP, YG, and JH assisted the experiments and provide some reagents. QW, YP, and YH wrote the manuscript. All authors discussed the results and commented on the manuscript.

## Conflict of Interest

The authors declare that the research was conducted in the absence of any commercial or financial relationships that could be construed as a potential conflict of interest.

## Footnotes

1 https://www.addgene.org/26214/

2 http://targetfinder.flycrispr.neuro.brown.edu/

## Supplementary Material

FIGURE S1 Alignment of human HYLS1, mice HYLS1 and *Drosophila* CG42231. Conserved amino acids are highlighted, and the conserved HYLS1 box is underlined. *Hs*: *Homo sapiens*; *Mm*: *Mus musculu*s; *Dm*: *Drosophila melanogaster*. Red * stands for the mutation site of hydrolethalus syndrome.

FIGURE S2 HYLS1 is a conserved centriole and basal body protein. **(A)** HYLS1 localizes to centrosomes in syncytial stage embryos. **(B)** Endogenous HYLS1 localizes to cilia base in sensory neurons. HYLS1 colocalizes with basal body marker γ-tubulin and is detected at the ciliary base of external sensory organ marked by 21A6. 21A6 indicates the cilium base. **(C)** Localization of endogenous HYLS1 in centriole and basal body during spermatogenesis. Blue channel in the merged panel represents DNA from mature spermatids. Bars: 2 μm.

FIGURE S3 Structural map of *hyls1* gene and *chiffon* gene. *chiffon* gene (CG5813, blue) overlaps with *hyls1* gene (CG42231, blue) and shares a common promoter, but differs in their open reading frames and codes different proteins. The coding sequences (black boxes), untranslated nucleotides (gray boxes), and introns (lines) are respectively illustrated to *chiffon* and *hyls1*. There are four annotated splice isoforms for *chiffon*: RA encodes a 1711-aa protein from two exons spanning a ∼1.1 kb intron; RB and RD encode 1695-aa proteins from a single ∼5 kb exon; RE encodes a 576-aa protein from a shorter exon within RB/RD. Deleted regions in *hyls1* and *chiffon-RA* are illustrated by black lines. gRNA editing to *hyls1* enabled the deletion of the last C-terminal 30 amino acids of CHIFFON-RA, including the stop codon (from 5043 bp to the 5136 bp).

FIGURE S4 Ultrastructure of the chordotonal cilia in WT and *hyls1* mutants. In WT, two axonemes are usually present in the cross section of a scolopidium. However, in *hyls1* mutants, missing axonemes is observed. Red arrow, normal axoneme. Green arrow, abnormal axoneme. Scale bars: 1 μm.

FIGURE S5 Quantification of IFT protein intensity relative to Actin intensity in the associated scolopidia in WT and *hyls1* mutants. Compare to WT, the ratios of IFT52 intensity/Actin intensity (control *n* = 35; *hyls1*^56^ *n* = 35), NOMPB intensity/Actin intensity (control *n* = 35; *hyls1*^56^ *n* = 26) and REMPA intensity/Actin intensity (control *n* = 31; *hyls1*^56^ *n* = 20) were significantly reduced in *hyls1* mutants. Error bars represent ± s.d., **p* < 0.05; ****p* ≤ 0.001 (Student’s *t-*test). “n” is the number of cilia examined.

TABLE S1 Primers used in this paper.

VIDEO S1 Heterozygous *hyls1^56^/Cyo* presented normal climbing activities.

VIDEO S2 *hyls1*^56^ null mutant flies showed climbing defect.

VIDEO S3 Uncoordinated phenotype in *hyls1*^56^ was rescued by introduction of a WT *hyls1* transgene in neurons driven by *elav*-GAL4.
