# Supplementary figures and images for: Functional Analysis of Hydrolethalus Syndrome Protein HYLS1 in Ciliogenesis and Spermatogenesis in Drosophila
Source: Front Cell Dev Biol. 2020 May 21;8:301. doi: 10.3389/fcell.2020.00301 (PMC7253586; doi:10.3389/fcell.2020.00301)

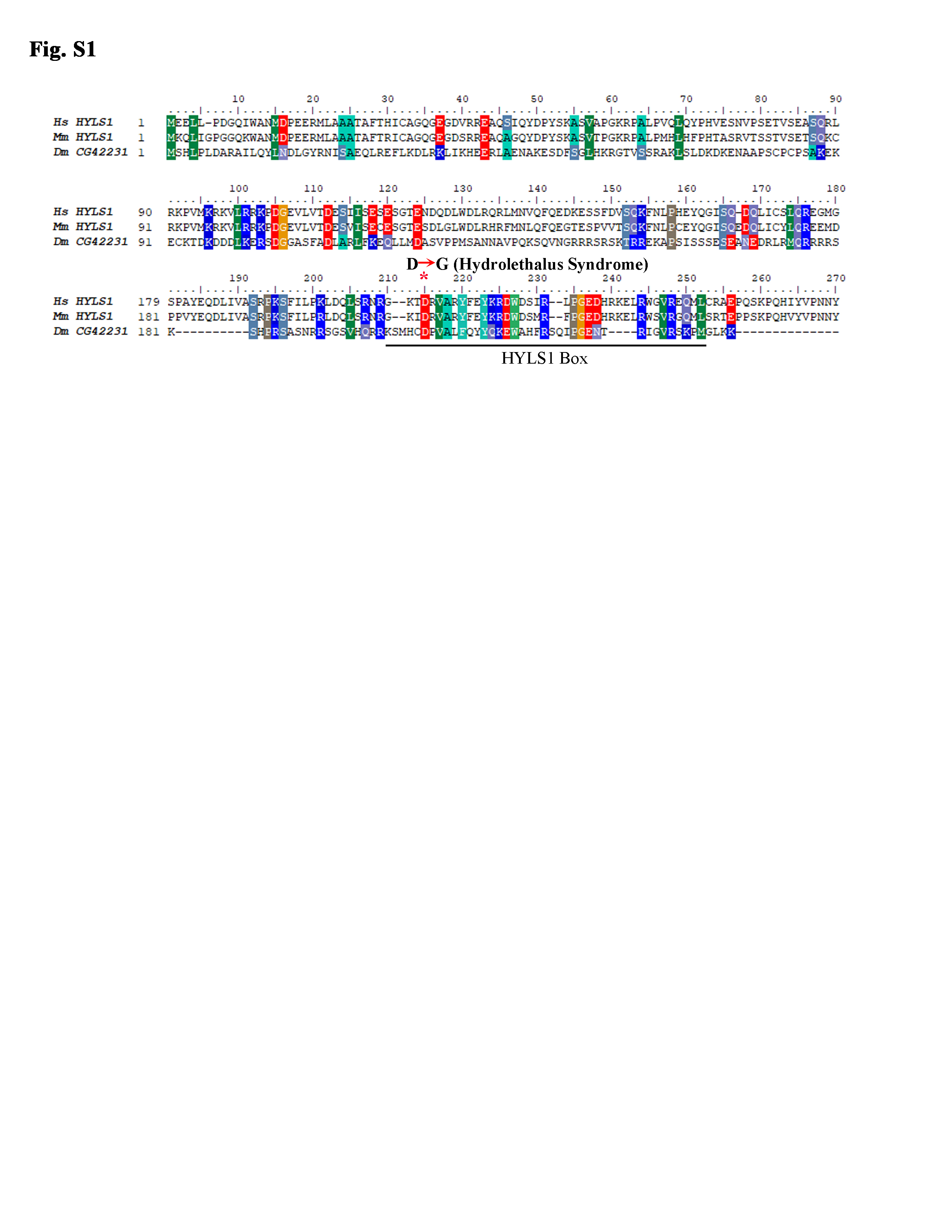

Supplement: FIGURE S1 — Alignment of human HYLS1, mice HYLS1 and Drosophila CG42231. Conserved amino acids are highlighted, and the conserved HYLS1 box is underlined. Hs: Homo sapiens; Mm: Mus musculus; Dm: Drosophila melanogaster. Red * stands for the mutation site of hydrolethalus syndrome. [file Image_1.jpg]

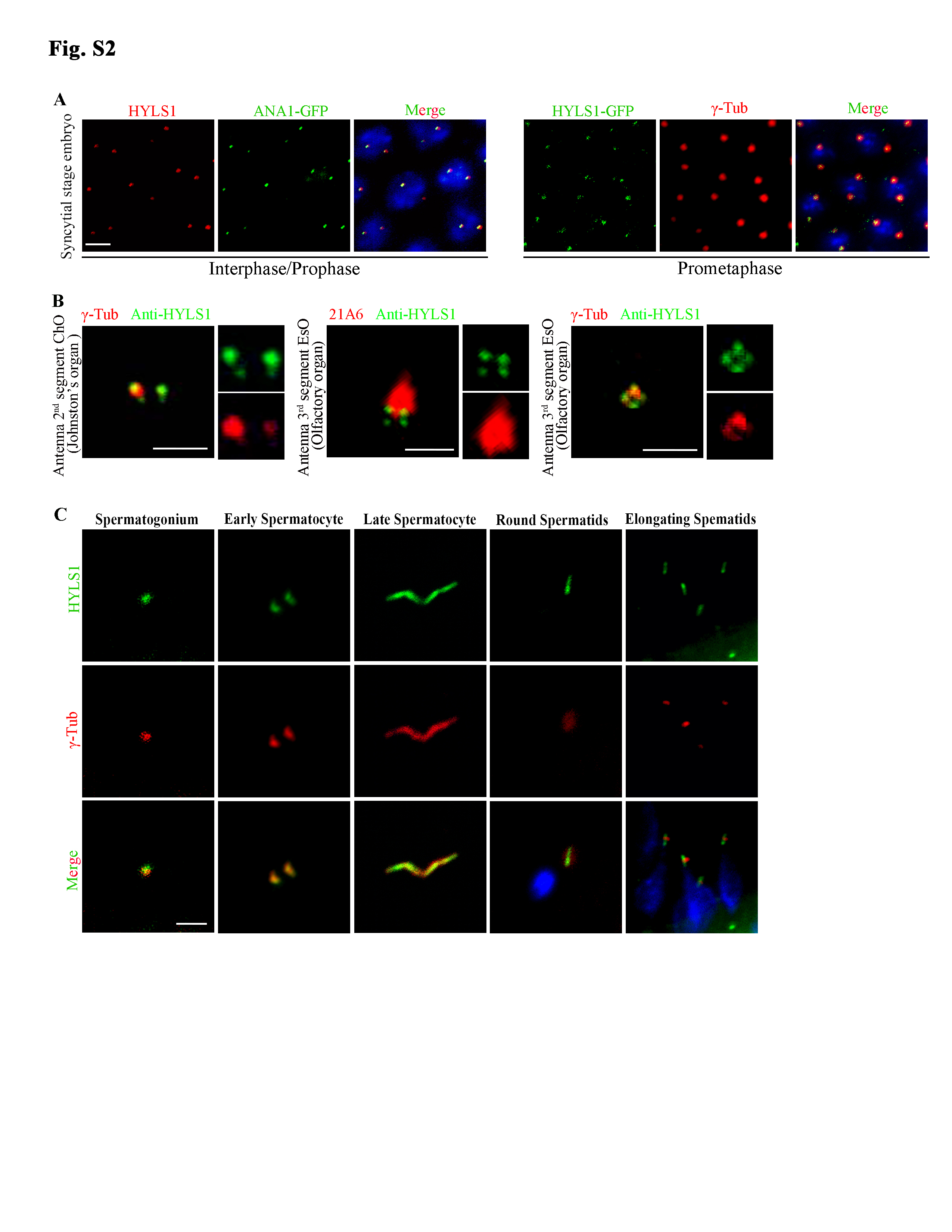

Supplement: FIGURE S2 — HYLS1 is a conserved centriole and basal body protein. (A) HYLS1 localizes to centrosomes in syncytial stage embryos. (B) Endogenous HYLS1 localizes to cilia base in sensory neurons. HYLS1 colocalizes with basal body marker γ-tubulin and is detected at the ciliary base of external sensory organ marked by 21A6. 21A6 indicates the cilium base. (C) Localization of endogenous HYLS1 in centriole and basal body during spermatogenesis. Blue channel in the merged panel represents DNA from mature spermatids. Bars: 2 μm. [file Image_2.jpg]

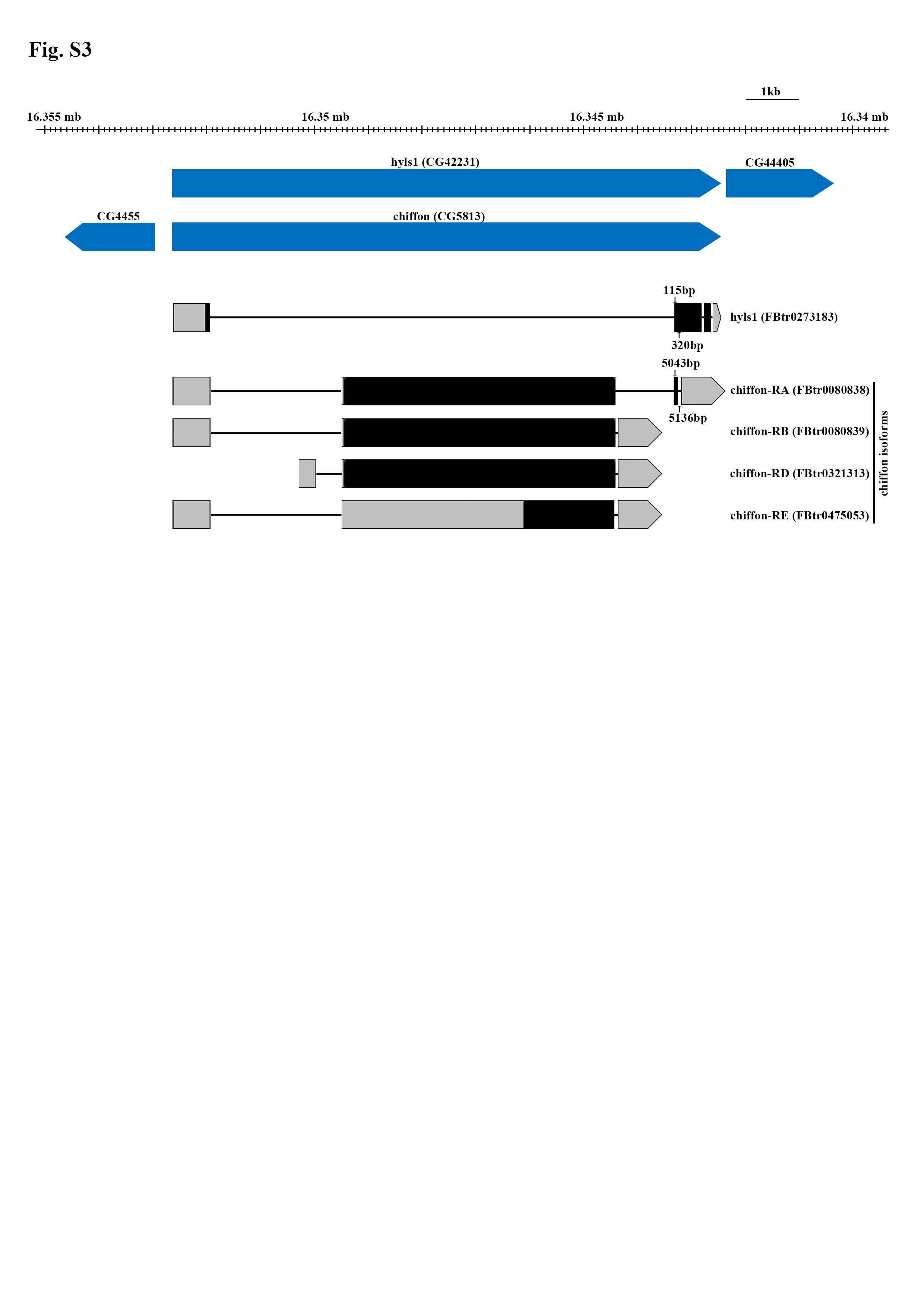

Supplement: FIGURE S3 — Structural map of hyls1 gene and chiffon gene. chiffon gene (CG5813, blue) overlaps with hyls1 gene (CG42231, blue) and shares a common promoter, but differs in their open reading frames and codes different proteins. The coding sequences (black boxes), untranslated nucleotides (gray boxes), and introns (lines) are respectively illustrated to chiffon and hyls1. There are four annotated splice isoforms for chiffon: RA encodes a 1711-aa protein from two exons spanning a ∼1.1 kb intron; RB and RD encode 1695-aa proteins from a single ∼5 kb exon; RE encodes a 576-aa protein from a shorter exon within RB/RD. Deleted regions in hyls1 and chiffon-RA are illustrated by black lines. gRNA editing to hyls1 enabled the deletion of the last C-terminal 30 amino acids of CHIFFON-RA, including the stop codon (from 5043 bp to the 5136 bp). [file Image_3.jpg]

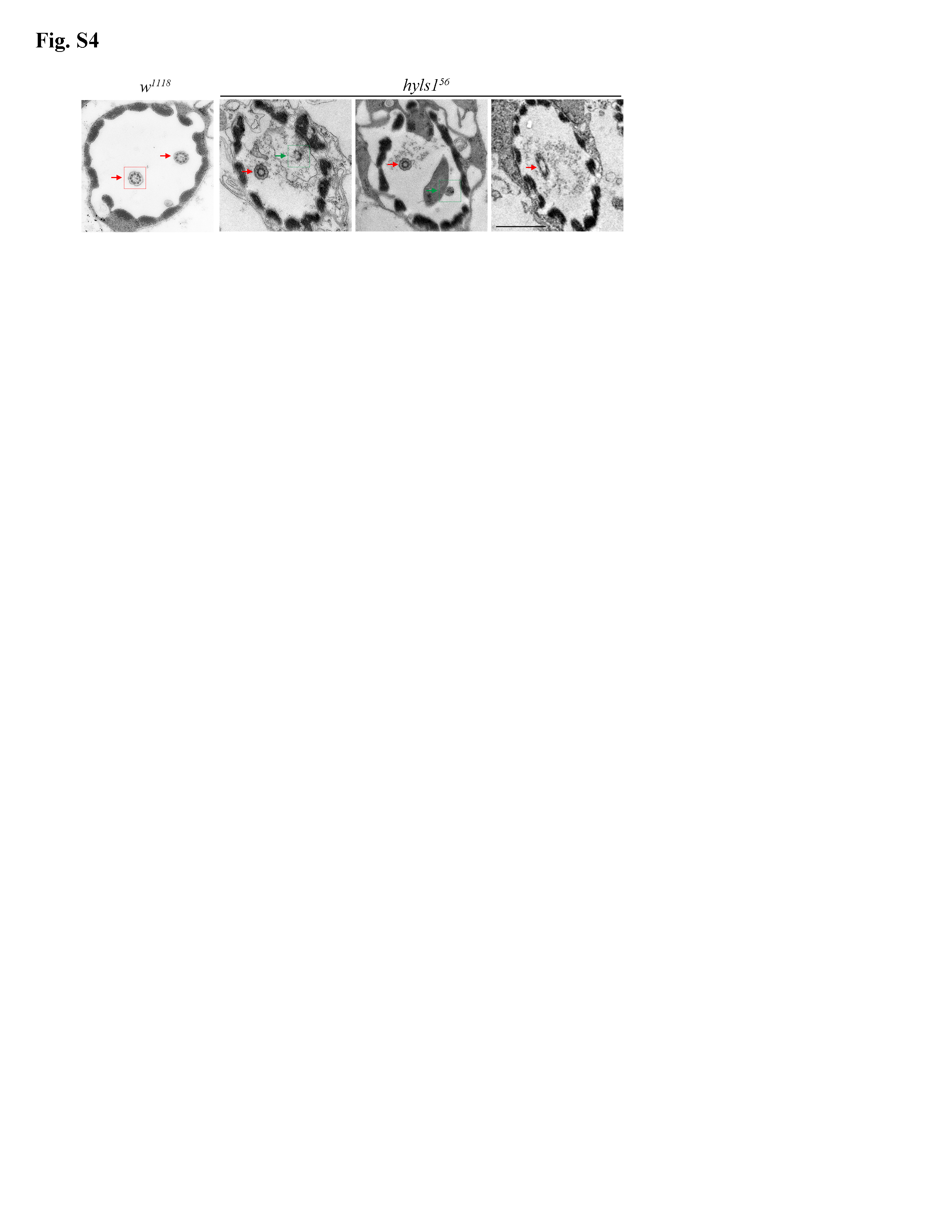

Supplement: FIGURE S4 — Ultrastructure of the chordotonal cilia in WT and hyls1 mutants. In WT, two axonemes are usually present in the cross section of a scolopidium. However, in hyls1 mutants, missing axonemes is observed. Red arrow, normal axoneme. Green arrow, abnormal axoneme. Scale bars: 1 μm. [file Image_4.jpg]

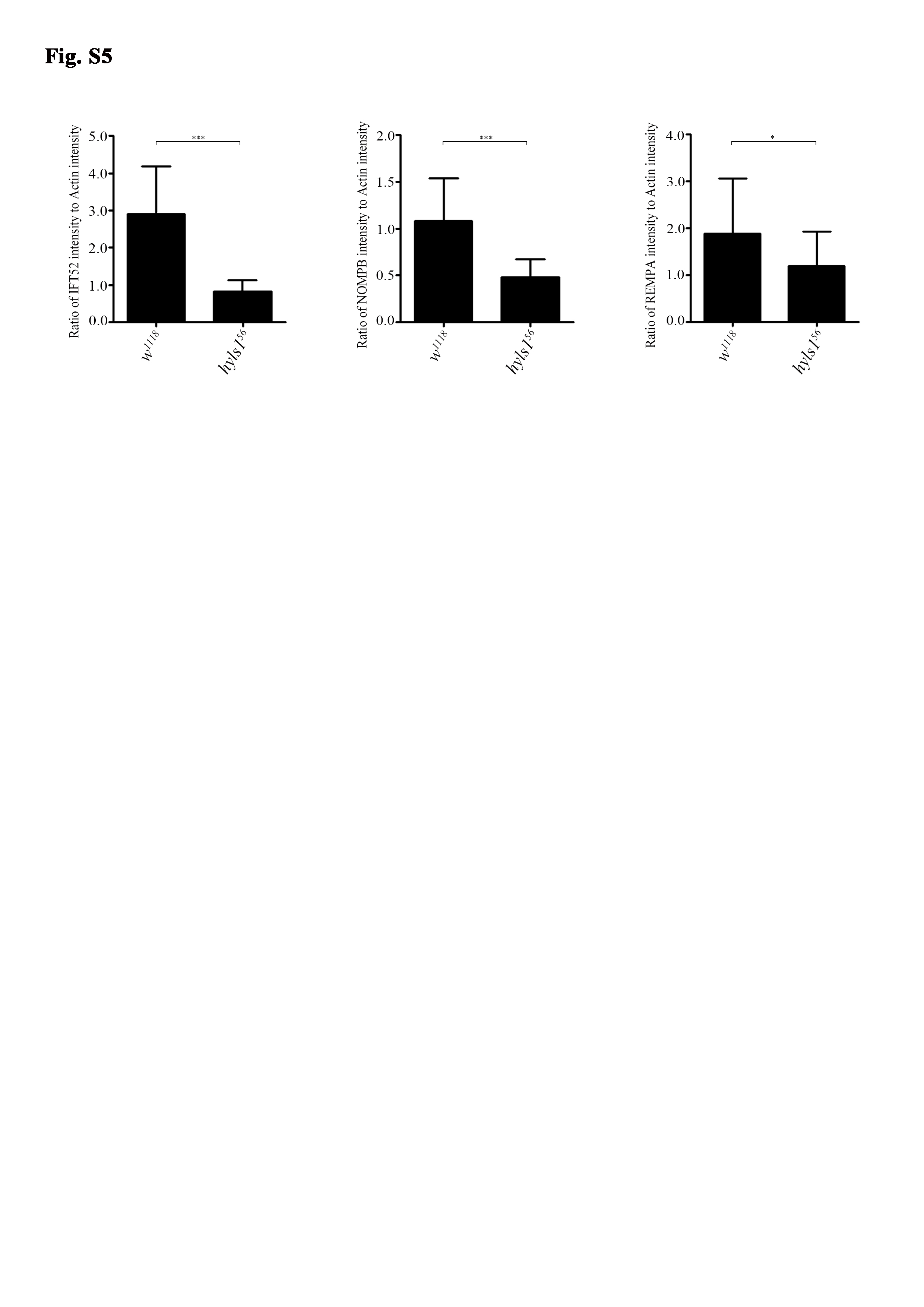

Supplement: FIGURE S5 — Quantification of IFT protein intensity relative to Actin intensity in the associated scolopidia in WT and hyls1 mutants. Compare to WT, the ratios of IFT52 intensity/Actin intensity (control n = 35; hyls156 n = 35), NOMPB intensity/Actin intensity (control n = 35; hyls156 n = 26) and REMPA intensity/Actin intensity (control n = 31; hyls156 n = 20) were significantly reduced in hyls1 mutants. Error bars represent ± s.d., *p < 0.05; ***p ≤ 0.001 (Student’s t-test). “n” is the number of cilia examined. [file Image_5.jpg]
