# Supplementary material for: Functional Analysis of Hydrolethalus Syndrome Protein HYLS1 in Ciliogenesis and Spermatogenesis in Drosophila
Source: Front Cell Dev Biol. 2020 May 21;8:301. doi: 10.3389/fcell.2020.00301 (PMC7253586; doi:10.3389/fcell.2020.00301)
Supplement: TABLE S1 — Primers used in this paper. [file Table_1.pdf]

Table S1. Primers used for transgenic constructs

Primer	Sequence
Ubq-hyls1-GFP forward	5'-CATTTTGCTCGAGCCGCGGCCGCATGTCACATTTGCCACTAGAT-3'
Ubq-hyls1-GFP reverse	5'-TTCTCCTTTACTCATGGATCCCTTTTTCAGGCCCATTGGCTTGCT-3'
pUAS-hyls1-GFP forward	5'-GAATGCGGCCGCATGTCACATTTGCCACTAGATGC-3'
pUAS-hyls1-GFP reverse	5'-CAAGGATCCCTTTTT CAGGCCCATTGGCTTGCTGCGTA-3'
Ubq-ana1-GFP forward	5'-CATTTTGCTCGAGCCGCGGCCGCATGGCTCTGCAGCTAACAGTA-3'
Ubq-ana1-GFP reverse	5'-TTCTCCTTTACTCATGGATCCTTTTCGGGGCGACTTTCGATG-3'
Ubq-cnn-GFP forward	5'-CATTTTGCTCGAGCCGCGGCCGCATGGACCAGTCTAAACAGGTTTTGCGG-3'
Ubq-cnn-GFP reverse	5'-TTCTCCTTTACTCATGGATCCTAACTCATTCTCCATGTTTGAGCGAACG-3'
Ubq-poc1-GFP forward	5'-CATTTTGCTCGAGCCGCGGCCGCATGCAAGGACTGTTCAGGGAT-3'
Ubq-poc1-GFP reverse	5'-TTCTCCTTTACTCATGGATCCAAAGAAGCGCGGACTGGAGCTATG-3'
fbf1-GFP forward	5'-CGTGGCCAGGGCCGCAAGCTTTATATCCCACCTTGACTGTCC -3'
fbf1-GFP reverse	5'-TTCTCCTTTACTCATGGATCCTATGAAGAATCTATCATTTCG-3'
unc-GFP forward	5'- CATTTTGCTCGAGCCGCGGCCGCATGAAGGTTCGCCACGTGC-3'
*unc-GFP r*everse	5'- TTCTCCTTTACTCATGGATCCCAGGTTTATGCGTTTCCAGAACG-3'
ift52-GFP forward	5'- CGTGGCCAGGGCCGCAAGCTTCTTCTCCACCAGCTTGAACCAATG -3'
ift52-GFP reverse	5'- TTCTCCTTTACTCATGGATCCGTCCTCCGCATAAGGTCTGTAGCT -3'
hyls1 mutant test forward	5'-ATCCACTATCCGCTATCCTCAT-3'
hyls1 mutant test reverse	5'-CTTTTTCAGGCCCATTGGCTTGCTGCGTA-3'
